# Characterization and Comparative Analysis of the Complete Plastomes of Five *Epidendrum* (Epidendreae, Orchidaceae) Species

**DOI:** 10.3390/ijms241914437

**Published:** 2023-09-22

**Authors:** Zhuang Zhao, Meng-Yao Zeng, Yu-Wei Wu, Jin-Wei Li, Zhuang Zhou, Zhong-Jian Liu, Ming-He Li

**Affiliations:** 1 Key Laboratory of National Forestry and Grassland Administration for Orchid Conservation and Utilization at College of Landscape Architecture and Art, Fujian Agriculture and Forestry University, Fuzhou 350002, China; fafuzzhuang@163.com (Z.Z.);; 2 Zhejiang Institute of Subtropical Crops, Zhejiang Academy of Agricultural Sciences, Wenzhou 325005, China; 3 Fujian Colleges and Universities Engineering Research Institute of Conservation and Utilization of Natural Bioresources, Fujian Agriculture and Forestry University, Fuzhou 350002, China

**Keywords:** *Epidendrum*, plastid genome, mutational hotspots, phylogenetic analysis

## Abstract

*Epidendrum*, one of the three largest genera of Orchidaceae, exhibits significant horticultural and ornamental value and serves as an important research model in conservation, ecology, and evolutionary biology. Given the ambiguous identification of germplasm and complex evolutionary relationships within the genus, the complete plastome of this genus (including five species) were firstly sequenced and assembled to explore their characterizations. The plastomes exhibited a typical quadripartite structure. The lengths of the plastomes ranged from 147,902 bp to 150,986 bp, with a GC content of 37.16% to 37.33%. Gene annotation revealed the presence of 78–82 protein-coding genes, 38 tRNAs, and 8 rRNAs. A total of 25–38 long repeats and 130–149 SSRs were detected. Analysis of relative synonymous codon usage (RSCU) indicated that leucine (Leu) was the most and cysteine (Cys) was the least. The consistent and robust phylogenetic relationships of *Epidendrum* and its closely related taxa were established using a total of 43 plastid genomes from the tribe Epidendreae. The genus *Epidendrum* was supported as a monophyletic group and as a sister to *Cattleya*. Meanwhile, four mutational hotspots (*trnC^GCA^*–*petN*, *trnD^GUC^*–*trnY^GUA^*, *trnS^GCU^*–*trnG^UCC^*, and *rpl32*–*trnL^UAG^*) were identified for further phylogenetic studies. Our analysis demonstrates the promising utility of plastomes in inferring the phylogenetic relationships of *Epidendrum*.

## 1. Introduction

Orchidaceae is recognized as one of the largest families of angiosperms, encompassing approximately 736 genera and 28,000 species [1,2]. *Epidendrum* L., a genus of the subtribe Lealiinae (tribe Epidendreae), is frequently celebrated as an outstanding example of adaptive radiation in vascular plants. It is among the largest genera of flowering plants, with approximately 1500 species that are mainly distributed in tropical and subtropical America [3]. *Epidendrum* species are almost epiphytic and caespitose, and their stems typically exhibit a cane-like morphology, while their petioles are characterized by tubular sheathing [3]. They possess significant horticultural and ornamental value due to the rich variations in the colors and shapes of their flowers. *Epidendrum* flowers are popular wedding flowers for bouquets, decorations, boutonnieres, and table centerpieces, as well as a favorite choice for prom corsages [4]. Meanwhile, the leaves of certain species within this genus have been traditionally utilized to address various health concerns, such as kidney problems, influenza, conjunctivitis, and liver pain, alleviating kidney symptoms and exhibiting a hypoglycemic effect [5]. This genus, similar to other Orchidaceae, exhibits a higher level of endangerment compared to other angiosperms [6]. Additionally, it demonstrates notable occurrences of natural hybridization and ecological interactions, rendered as a crucial subject for conservation biology, evolution, and ecology research [7,8,9,10,11,12].

The genus exhibits remarkable morphological diversification, characterized by a wide range of ancestral traits that pose challenges in an endeavor to delineate the generic circumscription [13]. Some morphological discussions have been conducted, including assessments of pseudobulb presence, column foot, and pollinia characteristics [14,15,16]. However, these investigations yielded uncompelling results. Van den Berg et al. first utilized the nuclear ribosomal ITS sequence to explore the phylogeny of Laeliinae, including 15 *Epidendrum* species, and the results indicated that *Epidendrum* was polyphyletic [17]. With the advancement of phylogenetic investigations on *Epidendrum*, incorporating multiple molecular fragment markers and expanding the taxonomic sampling range [1,3,11,18,19], a consensus of polyphyletic was reached regarding the delimitation of the genus. Meanwhile, the interrelationships remain incompletely resolved due to unstable topology and low support values.

Plastids play a crucial role in sustaining life on Earth by facilitating the conversion of solar energy into carbohydrates through photosynthesis and releasing oxygen. The plastid genome (plastome) has diverse applications in horticultural breeding, crop domestication, and phylogenetic studies [20]. The distinct genetic background, straightforward structure, and ability to provide substantial information regarding site characteristics make the plastome widely utilized in Orchidaceae phylogeny. Givnish et al. sampled 39 representative species of Orchidaceae to construct a phylogenetic tree based on the plastome [21]. The results revealed 5 subfamilies, 18 tribes, and 22 subtribes with strong support of Orchidaceae. Similarly, Liu et al. conducted a phylogenetic analysis using plastomes of 53 species belonging to the *Cleisostoma*–*Gastrochilus* clade. The results strongly supported the subdivision of this clade into six subclades, representing a significant improvement over previous studies that relied on several molecular fragment markers [22]. Overall, considerable progress has been made in utilizing plastomes to elucidate phylogenetic relationships within Orchidaceae at both the tribe or subtribe level and the interspecific level of genera. Studies have also investigated the impact of plastome features on the alteration of physiological traits and lifestyle in orchids, with particular emphasis on the transformation of epiphytic characteristics and mycoheterotrophic adaptations [23,24]. Among the largest genera in the Orchidaceae, substantial sections of the plastomes of *Bulbophyllum* and *Dendrobium* have been published and researched [25,26]. However, no plastome of *Epidendrum* has been currently reported.

In this study, we sequenced, assembled, and annotated the complete plastomes of five *Epidendrum* species, with the aim of (1) characterizing and contrasting the complete plastomes of *Epidendrum*; (2) understanding the evolutionary pattern of the *Epidendrum* plastome; and (3) evaluating variations in high-variability sites and simple sequence repeats (SSRs) for accurate authentication of *Epidendrum* species. Our study will contribute to the expansion of the genomic resources available for Orchidaceae and provide critical information to support the identification and phylogenetic analysis of different *Epidendrum* species.

## 2. Results

### 2.1. Genome Features

**Figure 1:**
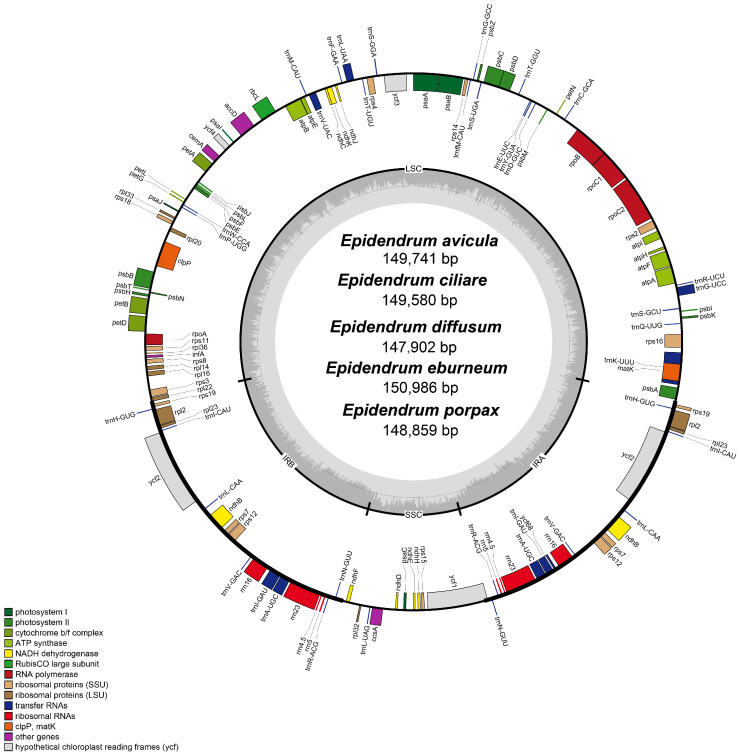
Annotation maps of five *Epidendrum* plastomes. The darker gray in the inner circle corresponds to the GC content. The IRa and IRb (two inverted repeating regions); LSC (large single-copy region); and SSC (small single-copy region) are indicated outside of the GC content.

The genome sizes of the five *Epidendrum* plastomes (Table 1) were determined to be 149,741 bp (*E. avicula*), 149,580 bp (*E. ciliare*), 147,902 bp (*E. diffusum*), 150,986 bp (*E. eburneum*), and 148,859 bp (*E. porpax*). The overall GC contents were found to be 37.16% (*E. avicula*), 37.33% (*E. ciliare*), 37.32% (*E. diffusum*), 37.19% (*E. eburneum*), and 37.27% (*E. porpax*). The plastomes exhibited a typical quadripartite structure (Figure 1). The length of the large single-copy (LSC) region ranged from 83,582 bp to 85,626 bp, with a GC content of 34.68% to 34.83%. The small single-copy (SSC) regions spanned from 11,059 bp to 12,659 bp, with a GC content of approximately 27.83% to 29.18%. The inverted repeat (IR) regions had a GC content of 41.23% to 41.41% and lengths ranging from 25,813 bp to 26,544 bp (Table 1).

The plastomes of *Epidendrum* encoded 38 transfer RNA (tRNA) genes and 8 ribosomal RNA (rRNA) genes. The plastome of *E. avicula* contained 129 genes, including 83 protein-coding genes; *E. ciliare* and *E. eburneum* contained 128 genes, including 82 protein-coding genes; *E. diffusum* contained 124 genes, including 78 protein-coding genes; and *E. porpax* contained 127 genes, including 81 protein-coding genes (Supplementary Table S1). The differences in gene numbers were due to the loss or pseudogenization of *ndh* genes (Supplementary Table S1). The plastomes of *E. ciliare* and *E. eburneum* encoded eight *ndh* genes (including two *ndh B* and one each of *ndh C*/*D*/*E*/*H*/*J*/*K*), *E. diffusum* encoded four *ndh* genes (including two *ndh B* and one each of *ndh E*/*H*), *E. porpax* encoded seven *ndh* genes (including two *ndh B* and one each of *ndh C*/*D*/*E*/*F*/*J*), and *E. avicula* encoded nine *ndh* genes (including two *ndh B* and one each of *ndh C*/*D*/*E*/*F*/*H*/*J*/*K*). The mauve visualization graphs indicated that the gene arrangement was conserved, and no significant gene rearrangement was detected among these plastomes (Supplementary Figure S1).

**Figure 2:**
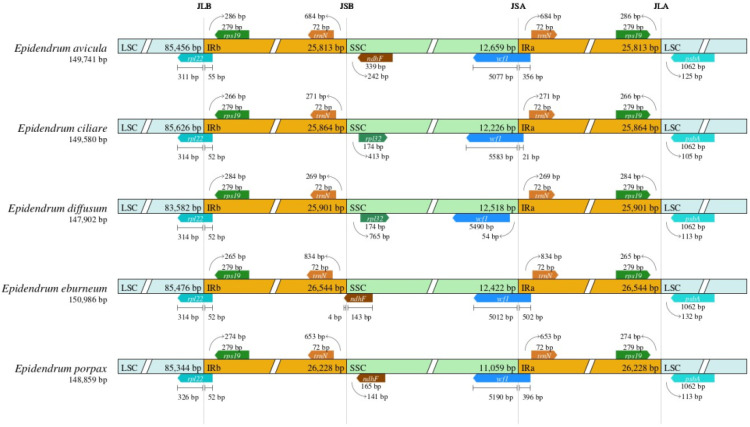
Comparison of the borders of LSC, SSC, and IR regions in the five *Epidendrum* species.

The IR boundary map was generated by comparing the plastomes of five *Epidendrum* species (Figure 2). The results showed that the *rps19* gene spanned the LSC/IRb boundary and primarily occurred in the LSC region, covering a length of 314 bp, with the remaining 52 bp located in the IRb region (*E. ciliare*, *E. diffusum*, *E. eburneum* and *E. porpax*), whereas in *E. avicula*, it comprised 311 bp situated in the LSC and 55 bp in the IRb region. For the IRb/SSC (JSB) region, the boundary was located on the right side of the *trnN^GUU^* region, and the distance from the *trnN^GUU^* to the JSB line ranged from 269 bp (*E. diffusum*) to 834 bp (*E. eburneum*). For the SSC/IRa (JSA) region, the *ycf1* gene spanned the SSC/IRa boundary and primarily occurred in the SSC region ranging from 5012 bp (*E. eburneum*) to 5583 bp (*E. ciliare*), whereas in *E. diffusum*, it was located on the right side of the JSA line and the distance was 54 bp. For the IRa/LSC (JLA) region, the *psbA* gene was located on the right side of the JLA line and the distance from the *psbA* to the JLA line ranged from 105 bp (*E. ciliare*) to 132 bp (*E. eburneum*).

### 2.2. Long Repeat and Simple Sequence Repeat (SSR) Analysis

**Figure 3:**
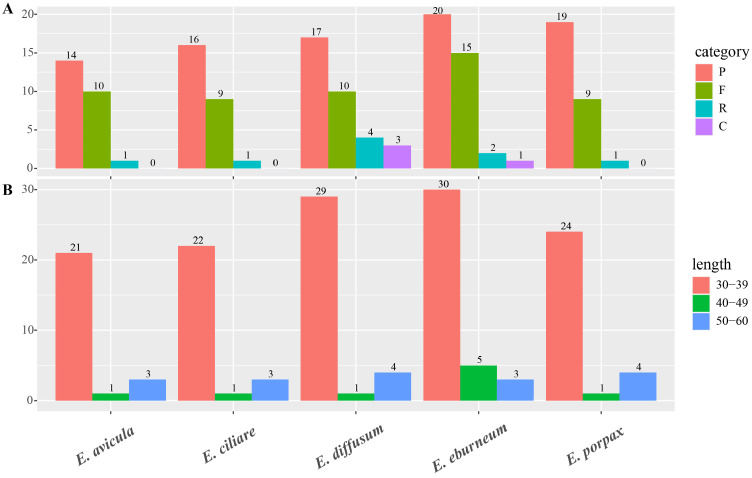
Comparison of long repeat sequences among five *Epidendrum* plastomes. (**A**) The number of each of four long repeat types (P, palindromic; F, forward; R, reverse; C, complement). (**B**) The number of long repeat sequences of different lengths.

In the analysis of the five *Epidendrum* plastomes, a total of 152 long repeat sequences were identified, encompassing four distinct categories: forward (F), reverse (R), complement (C), and palindromic (P) repeats (Figure 3A, Supplementary Table S2). Among them, all four categories were observed within two species (*E. diffusum* and *E. eburneum*), the other three species (*E. avicula*, *E. ciliare* and *E. porpax*) contained three categories of repeats (F, R, and P). The number of long repeat sequences varied in *E. avicula*, *E. ciliare*, *E. diffusum*, *E. eburneum,* and *E. porpax* with them containing 25, 26, 34, 38, and 29 repeats, respectively. Across these five genomes, palindromic repeats (P) were the most prevalent and ranged from 20 (*E. eburneum*) to 14 occurrences (*E. avicula*). R and C repeats of *E. eburneum* were less abundant and exhibited the highest counts of these types (4 and 3, respectively). The long repeat (30–39 bp) sequences were the most frequently observed and ranged from 21 (*E. avicula*) to 30 occurrences (*E. eburneum*). *E. eburneum* also displayed the highest count of 40–49 bp repeats (Figure 3B, Supplementary Table S3). The longest of the long repeat sequences was 57 bp (Supplementary Table S3).

**Figure 4:**
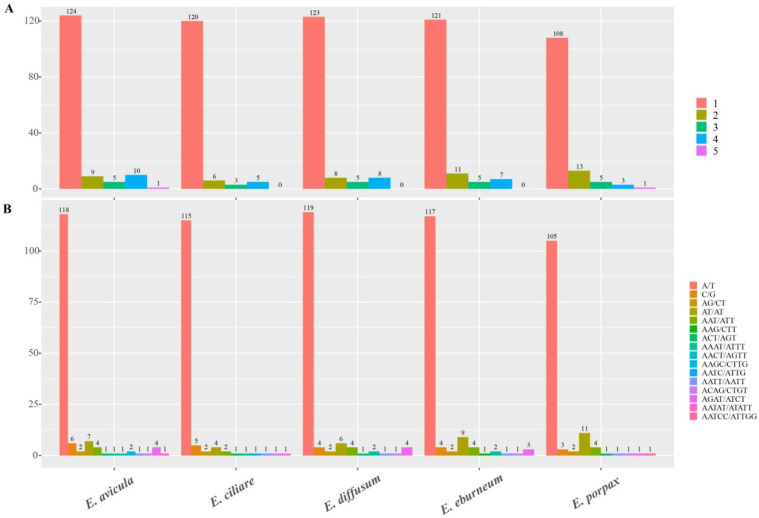
Comparison of simple sequence repeats (SSRs) among the five *Epidendrum* plastomes. (**A**) The number of SSRs containing one- to five-nucleotide motifs. (**B**) The number of different SSR motifs.

Simple sequence repeats (SSRs) composed of 1- to 6-nucleotide motifs were further examined. A total of 134, 144, 130, 144, and 149 SSRs were identified in the plastomes of *E. avicula*, *E. ciliare*, *E. diffusum*, *E. eburneum*, and *E. porpax*, respectively (Figure 4A, Supplementary Table S4). Mononucleotide SSRs were the most abundant type and accounted for 69.87%, while dinucleotide SSRs ranged from 6 (*E. ciliare*) to 13 occurrences (*E. porpax*). Tetranucleotide SSRs were more common than trinucleotide SSRs except for *E. porpax*, which exhibited the opposite trend. Pentanucleotide SSRs appeared only once in *E. porpax* and *E. avicula*, whereas hexanucleotide SSRs were absent from all genomes. Among the repeat motifs, the A/T mononucleotide motif was the most frequently observed, with it ranging from 108 (*E. porpax*) to 124 occurrences (*E. avicula*) (Figure 4B, Supplementary Table S4). The AT/AT dinucleotide motif was the second most prevalent and ranged from 4 (*E. ciliare*) to 11 occurrences (*E. porpax*). The C/G motifs appeared from 3 (*E. porpax*) to 6 occurrences (*E. avicula*) (Figure 4B, Supplementary Table S4).

### 2.3. Relative Synonymous Codon Usage Analysis

A total of 68 protein-coding genes were analyzed among the five *Epidendrum* plastomes, with the exception of the *ndh* genes due to incomplete gene loss and pseudogenization. These genes were encoded by a range of 19,305 (*E. diffusum*) to 19,405 (*E. eburneum*) codons (Table 2). The codon usage patterns are summarized in Table 2 and showed a highly conserved codon usage bias (CUB). Among them, one of the most frequent amino acids was leucine (Leu), appearing a total of 9751 times across all five plastomes. Reversely, cysteine (Cys) was the least frequent, occurring only 1062 times. Analysis of the relative synonymous codon usage (RSCU) indicated that GCU and CUU had the highest CUB, with average values of 1.866 and 1.725, respectively, whereas GCG and UAC had the lowest CUB, with average values of 0.364 and 0.374, respectively. Among the three stop codons, the frequency of UAA was the highest, accounting for 45.30%. The results also showed that 32 codons exhibited RSCU values greater than 1 and 30 codons exhibited less than 1. The RSCU values of AUG encoding for methionine (Met) and UGG encoding for threonine (Thr) were determined to be 1.

### 2.4. Sequence Variation and Barcoding Investigation

The divergence of the complete plastome sequences among the five *Epidendrum* species was analyzed using the mVISTA online platform, with *Stelis montserratii* as the reference. The results (Supplementary Figure S2) demonstrated that the full-length plastomes were highly conserved. The majority of the variable sites were observed in intergenic spacer regions. The coding regions exhibited greater conservation compared to the non-coding regions, and the IR regions were found to be more conserved than the LSC and SSC regions.

**Figure 5:**
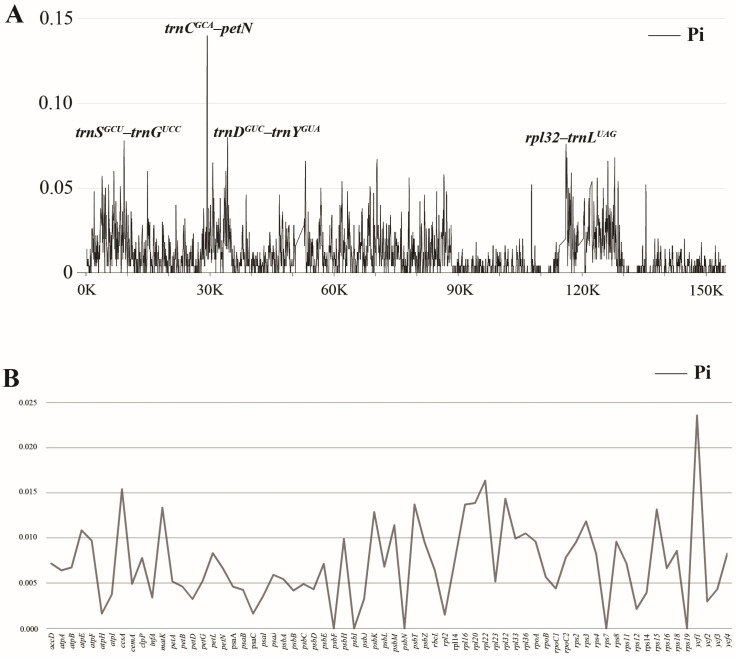
The nucleotide diversity (Pi) of *Epidendrum* plastomes and 68 protein-coding sequences. (**A**) For the nucleotide diversity of the complete plastome using a sliding window test, four mutation hotspot regions were annotated. The window size was set to 100 bp and the sliding windows size was 25 bp. *X*-axis, the position of the midpoint of a window; *Y*-axis, Pi values of each window. (**B**) The nucleotide diversity of 68 protein-coding sequences. *X*-axis, gene; *Y*-axis, Pi values of each gene.

To explore the highly mutable hotspots of *Epidendrum* plastomes, DnaSP6 was utilized to calculate nucleotide diversity (Pi). The results showed that the Pi values ranged from 0 to 0.14 (*trnC^GCA^*–*petN*) (Figure 5A, Supplementary Table S5). The IR regions exhibited the highest conservation with a value of 0.00342. The SSC region displayed the greatest nucleotide diversity (Pi = 0.02404), followed by the LSC region (Pi = 0.01294). According to the ranking of the Pi values, four hypervariable regions, including *trnC^GCA^*–*petN* (Pi = 0.14), *trnD^GUC^*–*trnY^GUA^* (Pi = 0.112), *trnS^GCU^*–*trnG^UCC^* (Pi = 0.088), and *rpl32*–*trnL^UAG^* (Pi = 0.08), were identified. Additionally, the nucleotide diversity of protein-coding genes was also analyzed. The results showed that the protein-coding genes have higher conservation (Figure 4B, Supplementary Table S6). Among these genes, *ycf1* (Pi = 0.02363), *rpl22* (Pi = 0.01639), *ccsA* (Pi = 0.01542), and *rpl32* (Pi = 0.01437) displayed the highest Pi values.

### 2.5. Phylogenetic Analysis

**Figure 6:**
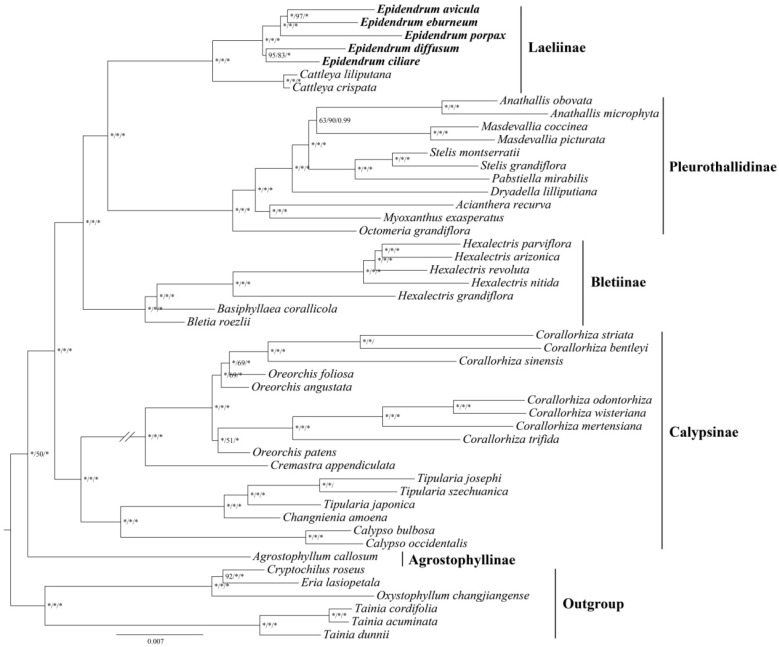
The phylogenetic tree of 43 Epidendreae species obtained by maximum-likelihood analysis based on complete plastome sequences. The numbers near the nodes are bootstrap percentages and Bayesian posterior probabilities (BP_ML_, BP_MP_, PP); * the node is the 100 bootstrap percentage or 1.00 posterior probability.

The phylogenetic analysis was conducted using complete plastome sequences and 68 protein-coding genes from 43 Epidendreae species (Figure 6, Supplementary Figure S3, Supplementary Table S7). The alignment matrix of complete plastome sequences was 207,121 bp, with 42,905 variable sites and 23,167 parsimony informative sites. The matrix of 68 protein-coding genes was 62,292 bp, with 10,746 variable sites and 57,000 parsimony informative sites. The results revealed a consistent topological structure, and the main clades were strongly supported (Figure 6, Supplementary Figure S3). Specifically, *Epidendrum* was robustly supported as a monophyletic group (BS = 100, PP = 1.00) and as a sister to *Cattleya*. Within the genus, the analyses collectively supported the following relationships: ((*E. ciliare*, *E. diffusum*) (*E. porpax* (*E. eburneum*, *E. avicula*))). Furthermore, the tribe phylogenetic tree unveiled that Epidendreae were recovered as a monophyletic group and the following relationships of five subtribes were unveiled: (Agrostophyllinae (Calypsinae (Bletiinae (Pleurothallidinae, Laeliinae)))).

## 3. Discussion

### 3.1. Plastome Characteristics and Structure

In this study, the complete plastomes (including five species) of *Epidendrum* were first reported and a total of five species were sequenced and assembled. This provides a valuable opportunity to gain further insights into the evolution of plastomes in this complex genus. Similar to most angiosperms, the *Epidendrum* plastome exhibited a typical quadripartite structure, consisting of one LSC, one SSC, and two IR regions. The genome size (ranging from 147,902 to 150,986 bp) and GC content (ranging from 37.16% to 37.33%) fall within the range of other orchids, such as the epiphytic orchids *Dendrobium*, *Holcoglossum*, and *Paraphalaenopsis* [26,27,28]. The five *Epidendrum* plastomes identified a total of 124 to 129 genes, consisting of 78 to 83 protein-coding genes, 38 tRNAs, and 8 rRNAs (Figure 1, Supplementary Table S1). The variations in gene content were revealed due to the incomplete loss of *ndh* genes, which was also found in other orchid species, such as *Dendrobium* and *Polystachya* [26,29]. These phenomena may potentially indicate a correlation with the epiphytic lifestyle [30]. Analyses of Mauve collinearity and plastome boundaries were conducted in this study (Supplementary Figure S1), which revealed a highly conserved structure among these plastomes. It is well-known that the contraction and expansion of the IR boundary are common during the evolution of plastids, significantly contributing to the variation in plastome length and gene content in angiosperms [31]. In terms of gene arrangement at the boundaries within the five *Epidendrum* plastomes (Figure 2), we observed a high degree of conservation at JLA and JLB. However, the variations of JSB (Figure 2) could be attributed to the loss of *ndhF* in *E. ciliare* and *E. diffusum*. 

The identification of long repeat sequences and SSRs within chloroplast genomes plays a crucial role in recognizing plant germplasm resources and molecular markers, making it an essential aspect of scientific research in this field [32]. For long repeat sequences (Figure 3), complement repeats were only observed in *E. diffusum* and *E. eburneum*. The other three repeat units were shared among all five plastomes, with slight differentiation in terms of the number of repeat units and their proportions. Simple-sequence repeats (SSRs) are short tandem repeat DNA sequences that consist of repeating 1–6 nucleotide motifs (Figure 4). These motifs are widely distributed throughout the plastomes and serve as crucial molecular markers for analyzing genetic diversity and species relationships [33]. In this study, a total of 134 to 149 SSRs were identified in the plastomes of *Epidendrum* (Figure 4). A/T SSRs were found to be more abundant compared to G/C SSRs, which is consistent with other orchids [29,34]. Additionally, the numbers of Dinucleotide SSRs varied across the five *Epidendrum* plastomes, which can be employed for further population genetic and phylogenetic research [32]. The relative synonymous codon usage (RSCU) is highly valuable in understanding the preference for synonymous codon usage [35]. The codon frequency and RSCU values displayed similar patterns in *Epidendrum* plastomes (Table 2). Among all codons, leucine (Leu) exhibited the highest occurrence, while cysteine (Cys) had the lowest frequency. These findings are consistent with previous studies on codon preference in Orchidaceae [27,33,36] and further demonstrate the high level of plastome conservation in *Epidendrum*.

### 3.2. Plastid Genomic Evolutionary Hotspots

Earlier studies have shown that the IR regions are highly conserved, and coding regions exhibit greater conservation compared to the non-coding regions in the plastomes of Orchidaceae [27,28,29,34,36]. The results of complete plastome divergence among *Epidendrum* (Figure 5) are consistent with previous findings. DNA barcoding can be employed as a method to identify species [37]. With advancements in molecular phylogenetics, DNA barcoding of Orchidaceae has successfully utilized *rbcL*, *matK*, *atpB*, *psaB*, *Xdh*, *trnL-F*, and ITS [38,39,40]. While these phylogenetics have established an initial framework for understanding the phylogeny of orchids, most taxonomic complexities, especially the intrageneric phylogenetic relationships, still pose various challenges. Currently, plastomes are commonly used in phylogenetic studies and species identification [41]. In this study, four hypervariable regions, including *trnC^GCA^*–*petN*, *trnD^GUC^*–*trnY^GUA^*, *trnS^GCU^*–*trnG^UCC^*, and *rpl32*–*trnL^UAG^*, were identified. Although four protein-coding genes, including *ycf1*, *rpl22*, *ccsA*, and *rpl32*, also displayed high Pi values, they still remain highly conserved. These results demonstrate that the aforementioned intergenic spacer regions are more suitable as candidate barcodes compared to protein-coding genes.

### 3.3. Phylogeny of Epidendrum and Its Related Taxon

The tribe Epidendreae was initially defined as a group of tropical epiphytic orchids, and the circumscription has constantly changed with the discovery of a large number of tropical orchids [42]. Based on molecular phylogenetics, the Epidendreae were supported and divided into five subtribes: Bletiinae, Chysiinae, Laeliinae, Ponerinae, and Pleurothallidinae [42]. Szlachetko proposed that Epidendreae consisted of Coellinae, Chysiinae, Meiracyllinae, Laellinae (including *Cattleya*), Epidendrinae (including *Epidendrum*), Ponerinae, and Pleurothallidinae based on morphological characteristics [43]. Among these subtribes, Epidendrinae and Laellinae were closest and had the same seed morphology but differed in gynostemium and anther. This classification was widely accepted by subsequent studies until Chase et al. [1]. In this study, the phylogenetic analysis based on 68 protein-coding genes and complete plastome sequences robustly supported the sister relationship between *Cattleya* and *Epidendrum*. Five monophyletic groups, Agrostophyllinae, Calypsoinae, Bletiinae, Pleurothallidinae, and Laeliinae, were also unveiled with high support (Figure 6, Supplementary Figure S3). These findings are consistent with previous studies and demonstrate the effectiveness of using plastomes to subtribe the phylogenetics of Epidendreae.

Van den Berg et al. proposed a hypothesis for the circumscription of the *Epidendrum* alliance based on ITS sequences. Subsequently, more samples and the *matK* fragment were added to further explore this alliance [2,17]. The findings revealed that the range of *Epidendrum* has been significantly underestimated. The phylogenetic analysis, conducted using several molecular fragments, indicated that the *Epidendrum* alliance consists of *Epidendrum*, *Caularthron*, *Orleanesia*, *Barkeria*, and *Myrmecophila*, with a close relationship with the *Laelia* alliance [18]. Considering the unstable topologies and low support rates [2,17,18], it is crucial to conduct further investigations into the phylogenetic relationships of *Epidendrum* and its related taxa. In certain groups within the Orchidaceae, which have undergone rapid radiation and consist of numerous species, plastomes have shown excellent performance in phylogenetic studies [23]. In this study, the phylogenetic analysis based on 68 protein-coding genes and complete plastomes demonstrated a stable topology and provided high support for five *Epidendrum* species (Figure 5, Supplementary Figure S3). The results demonstrate the power of plastid phylogenomics to improve the phylogenetic relationships of *Epidendrum*.

## 4. Materials and Methods

### 4.1. Sample Sampling, DNA Extraction and Sequencing, Plastome Assembly and Annotation

In this study, we sequenced five *Epidendrum* species, and their voucher specimens were stored at the herbarium of the College of Forestry, Fujian Agriculture and Forestry University (FJFC). A total of 49 plastomes from 24 genera were selected, including 4 genera with 6 species designated as outgroups based on the classification of Chase et al. [1]. Supplementary Table S7 provides details on the taxa, including voucher information and GenBank accession numbers. To perform DNA extraction, sequencing, plastome assembly, and annotation, we followed the protocols outlined in our previous study [23].

### 4.2. Analysis of Plastome Structure, Repeat Sequences, and Codon Usage

To identify rearrangements among different *Epidendrum* plastomes, we utilized the software Mauve (http://gel.ahabs.wisc.edu/mauve, accessed on 22 August 2023) [44]. The online R Shiny application CPJSdraw 0.0.1 [45] was employed to visually analyze and evaluate the differences in the boundaries of the LSC/IR/SSC regions across all five *Epidendrum* plastomes. Using REPuter (https://bibiserv.cebitec.uni-bielefeld.de/reputer, accessed on 22 August 2023) [46] with default parameters, we detected four types of long repeats (F—forward, P—palindrome, R—reverse, and C—complement) in the five plastomes. Simple sequence repeats (SSRs) were detected using the Perl script MISA [47], with the minimum thresholds for mono-, di-, tri-, tetra-, penta-, and hexa-motif microsatellites set at 10, 5, 4, 3, 3, and 3 nucleotide repeats, respectively. The R package ggplot2 [48] was used to visualize the results.

To extract the 68 protein-coding genes from the five *Epidendrum* plastomes, we employed PhyloSuite v1.2.2 [49]. These genes were then concatenated using Phylosuite v1.2.2, and their relative synonymous codon usage (RSCU) for each *Epidendrum* species was analyzed using DAMBE7 [50].

### 4.3. Analysis of Sequence Variation, Barcoding Investigation, and Phylogeny

To analyze the diversity of *Epidendrum* plastome sequences, the online program mVISTA (https://genome.lbl.gov/vista/mvista, accessed on 22 August 2023) was utilized, employing the Shuffle-LAGAN alignment program [51]. The plastome of *Stelis montserratii* (MW375125) was used as the reference. Nucleotide variability (Pi) for the five *Epidendrum* plastomes and 68 protein-coding genes of the plastome was calculated using DnaSP 6 [52] with a window length of 100 bp and a step size of 25 bp.

For phylogenetic tree construction, a concatenated matrix of the 68 protein-coding genes and a matrix of complete plastomes were used. The 68 protein-coding genes were extracted and concatenated using PhyloSuite v1.2.2 [50]. The complete plastomes and the concatenated 68 protein-coding genes were aligned using MAFFT v7.471 [53]. Phylogenetic relationships were then analyzed using maximum parsimony (MP), maximum likelihood (ML), and Bayesian inference (BI), following the protocols described in our previous study [23].

## 5. Conclusions

In this study, we firstly report the complete plastomes (including five species) of *Epidendrum*, and a total of five species were sequenced and assembled. Our findings reveal a high degree of conservation in terms of sequence lengths, boundaries of inverted repeats (IR), repeat sequences, and codon usage among *Epidendrum* plastomes. Moreover, we have identified certain regions (*trnC^GCA^*–*petN*, *trnD^GUC^*–*trnY^GUA^*, *trnS^GCU^*–*trnG^UCC^*, and *rpl32*–*trnL^UAG^*) as mutational hotspots, which hold potential for further phylogenetic investigations. By employing phylogenomic analysis, we have demonstrated that plastomes offer valuable insights into the phylogenetic relationships within *Epidendrum*. These findings underscore the significance of plastomes as a robust tool for studying the evolutionary history of *Epidendrum*. Overall, our study contributes to the expanding library of sequenced plastomes in *Epidendrum*. Future research can capitalize on these findings to deepen our understanding of the plastome evolutionary relationships within *Epidendrum* and related taxa.

## Figures and Tables

**Table 1:** Complete plastome features of *Epidendrum*.

Feathers	*E. avicula*	*E. ciliare*	*E. diffusum*	*E. eburneum*	*E. porpax*
Total length (bp)	149,741	149,580	147,902	150,986	148,859
GC content (%)	37.16	37.33	37.32	37.19	37.27
LSC length (bp)	85,456	85,626	83,582	85,476	85,344
GC content (%)	34.72	34.83	34.80	34.68	34.72
SSC length (bp)	12,659	12,226	12,518	12,422	11,059
GC content (%)	28.32	29.18	28.93	28.65	27.83
IR length (bp)	25,813	25,864	25,901	26,544	26,228
GC content (%)	43.37	43.41	43.41	43.23	43.40
Number of genes	129	128	124	128	127
Protein-coding genes	83	82	78	82	81
tRNA genes	38	38	38	38	38
rRNA genes	8	8	8	8	8
*ndh* genes	9	8	4	8	7
Specimen voucher	MHLi or147	MHLi or123	MHLi or137	MHLi or140	MHLi or131
Accession number	OR460026	OR460022	OR460023	OR460025	OR460024

**Table 2:** The relative synonymous codon usage (RSCU) values of all 64 codons for the five *Epidendrum* plastomes. * indicates stop codon.

Codon	AA	*E. avicula*		*E. ciliare*		*E. diffusum*		*E. eburneum*		*E. porpax*	
		Frequency	RSCU	Frequency	RSCU	Frequency	RSCU	Frequency	RSCU	Frequency	RSCU
UAA	*	32	1.412	30	1.324	29	1.279	31	1.368	32	1.412
UAG	*	17	0.750	19	0.838	19	0.838	19	0.838	19	0.838
UGA	*	19	0.838	19	0.838	20	0.882	18	0.794	17	0.750
GCA	Ala	321	1.224	320	1.220	322	1.221	323	1.234	322	1.233
GCC	Ala	145	0.553	144	0.549	140	0.531	141	0.539	143	0.547
GCG	Ala	93	0.355	97	0.370	99	0.375	94	0.359	94	0.360
GCU	Ala	490	1.868	488	1.861	494	1.873	489	1.868	486	1.860
UGC	Cys	55	0.524	51	0.474	53	0.495	52	0.491	54	0.512
UGU	Cys	155	1.476	164	1.526	161	1.505	160	1.509	157	1.488
GAC	Asp	148	0.376	149	0.376	152	0.386	149	0.375	147	0.373
GAU	Asp	639	1.624	643	1.624	635	1.614	645	1.625	641	1.627
GAA	Glu	821	1.519	832	1.524	822	1.522	839	1.532	828	1.523
GAG	Glu	260	0.481	260	0.476	258	0.478	256	0.468	259	0.477
UUC	Phe	376	0.701	379	0.704	382	0.712	379	0.703	376	0.697
UUU	Phe	696	1.299	697	1.296	691	1.288	699	1.297	703	1.303
GGA	Gly	507	1.543	506	1.536	507	1.542	510	1.547	515	1.561
GGC	Gly	130	0.396	133	0.404	130	0.395	133	0.403	128	0.388
GGG	Gly	236	0.718	239	0.725	236	0.718	233	0.707	232	0.703
GGU	Gly	441	1.342	440	1.335	442	1.344	443	1.343	445	1.348
CAC	His	110	0.448	110	0.451	111	0.452	110	0.453	110	0.451
CAU	His	381	1.552	378	1.549	380	1.548	376	1.547	378	1.549
AUA	Iso	473	0.880	492	0.909	483	0.898	485	0.902	484	0.900
AUC	Iso	332	0.618	335	0.619	327	0.608	332	0.617	333	0.619
AUU	Iso	807	1.502	797	1.472	803	1.493	796	1.480	797	1.481
AAA	Lys	826	1.495	823	1.484	814	1.485	827	1.486	819	1.470
AAG	Lys	279	0.505	286	0.516	282	0.515	286	0.514	295	0.530
CUA	Leu	255	1.123	264	1.140	263	1.142	261	1.129	271	1.172
CUC	Leu	125	0.551	127	0.549	124	0.539	126	0.545	122	0.528
CUG	Leu	136	0.599	137	0.592	134	0.582	137	0.592	137	0.592
CUU	Leu	392	1.727	398	1.719	400	1.737	401	1.734	395	1.708
UUA	Leu	617	1.198	612	1.192	609	1.183	616	1.194	615	1.198
UUG	Leu	413	0.802	415	0.808	421	0.817	416	0.806	412	0.802
AUG	Met	439	1.000	438	1.000	436	1.000	446	1.000	439	1.000
AAC	Asp	192	0.413	188	0.409	190	0.410	191	0.411	193	0.420
AAU	Asp	737	1.587	731	1.591	736	1.590	738	1.589	725	1.580
CCA	Pro	211	1.098	213	1.095	211	1.090	212	1.091	208	1.081
CCC	Pro	174	0.905	174	0.895	175	0.904	176	0.906	173	0.899
CCG	Pro	85	0.442	84	0.432	85	0.439	84	0.432	86	0.447
CCU	Pro	299	1.555	307	1.578	303	1.566	305	1.570	303	1.574
CAA	Glu	568	1.548	565	1.546	564	1.547	569	1.546	571	1.550
CAG	Glu	166	0.452	166	0.454	165	0.453	167	0.454	166	0.450
AGA	Arg	376	1.510	388	1.525	385	1.513	384	1.509	383	1.520
AGG	Arg	122	0.490	121	0.475	124	0.487	125	0.491	121	0.480
CGA	Arg	275	1.558	269	1.528	272	1.541	271	1.535	271	1.542
CGC	Arg	64	0.363	67	0.381	67	0.380	67	0.380	65	0.370
CGG	Arg	82	0.465	81	0.460	81	0.459	81	0.459	82	0.467
CGU	Arg	285	1.615	287	1.631	286	1.620	287	1.626	285	1.622
AGC	Ser	80	0.409	78	0.394	79	0.406	78	0.397	79	0.403
AGU	Ser	311	1.591	318	1.606	310	1.594	315	1.603	313	1.597
UCA	Ser	287	1.073	288	1.076	283	1.062	284	1.059	290	1.074
UCC	Ser	230	0.860	227	0.848	224	0.841	231	0.861	232	0.859
UCG	Ser	109	0.407	112	0.418	113	0.424	115	0.429	111	0.411
UCU	Ser	444	1.660	444	1.658	446	1.674	443	1.651	447	1.656
ACA	Thr	296	1.221	298	1.214	294	1.212	306	1.250	301	1.229
ACC	Thr	177	0.730	173	0.705	175	0.722	172	0.703	179	0.731
ACG	Thr	105	0.433	104	0.424	104	0.429	100	0.409	101	0.412
ACU	Thr	392	1.616	407	1.658	397	1.637	401	1.638	399	1.629
GUA	Val	385	1.454	386	1.464	385	1.462	384	1.449	386	1.458
GUC	Val	135	0.510	132	0.500	133	0.505	135	0.509	131	0.495
GUG	Val	159	0.601	160	0.607	158	0.600	160	0.604	164	0.619
GUU	Val	380	1.435	377	1.429	377	1.432	381	1.438	378	1.428
UGG	Try	340	1.000	340	1.000	336	1.000	337	1.000	336	1.000
UAC	Tyr	126	0.370	124	0.368	128	0.383	128	0.378	125	0.370
UAU	Tyr	556	1.630	550	1.632	540	1.617	550	1.622	551	1.630

## Data Availability

The five newly obtained complete plastid genome sequences that support the findings of the study have been deposited in the NCBI with accession numbers as followes: OR460022-OR460026.

## Supplementary Materials

The following supporting information can be downloaded at: https://www.mdpi.com/article/10.3390/ijms241914437/s1.
