# Supplementary material for: Characterization and Comparative Analysis of the Complete Plastomes of Five Epidendrum (Epidendreae, Orchidaceae) Species
Source: Int J Mol Sci. 2023 Sep 22;24(19):14437. doi: 10.3390/ijms241914437 (PMC10572996; doi:10.3390/ijms241914437)
Supplement: Supplementary file 1 [file ijms-24-14437-s001.zip › 0822_Supplementary Figures.pdf]

Supplementary Figures

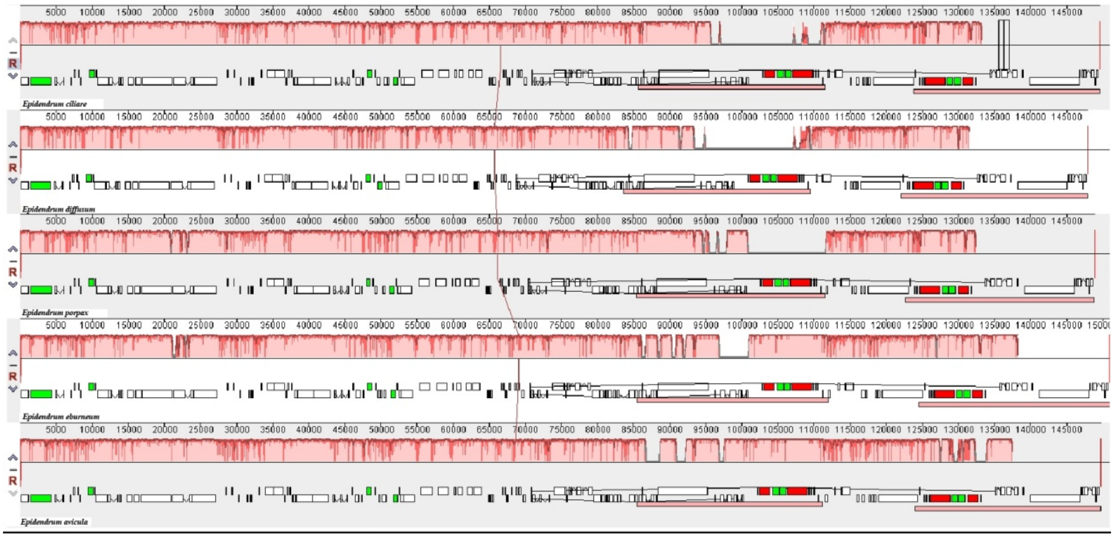

Supplementary Figure S1 Alignment of the five *Epidendrum* plastomes (mauve graphs). Local collinear blocks within each alignment are represented by blocks of the same color connected with lines.

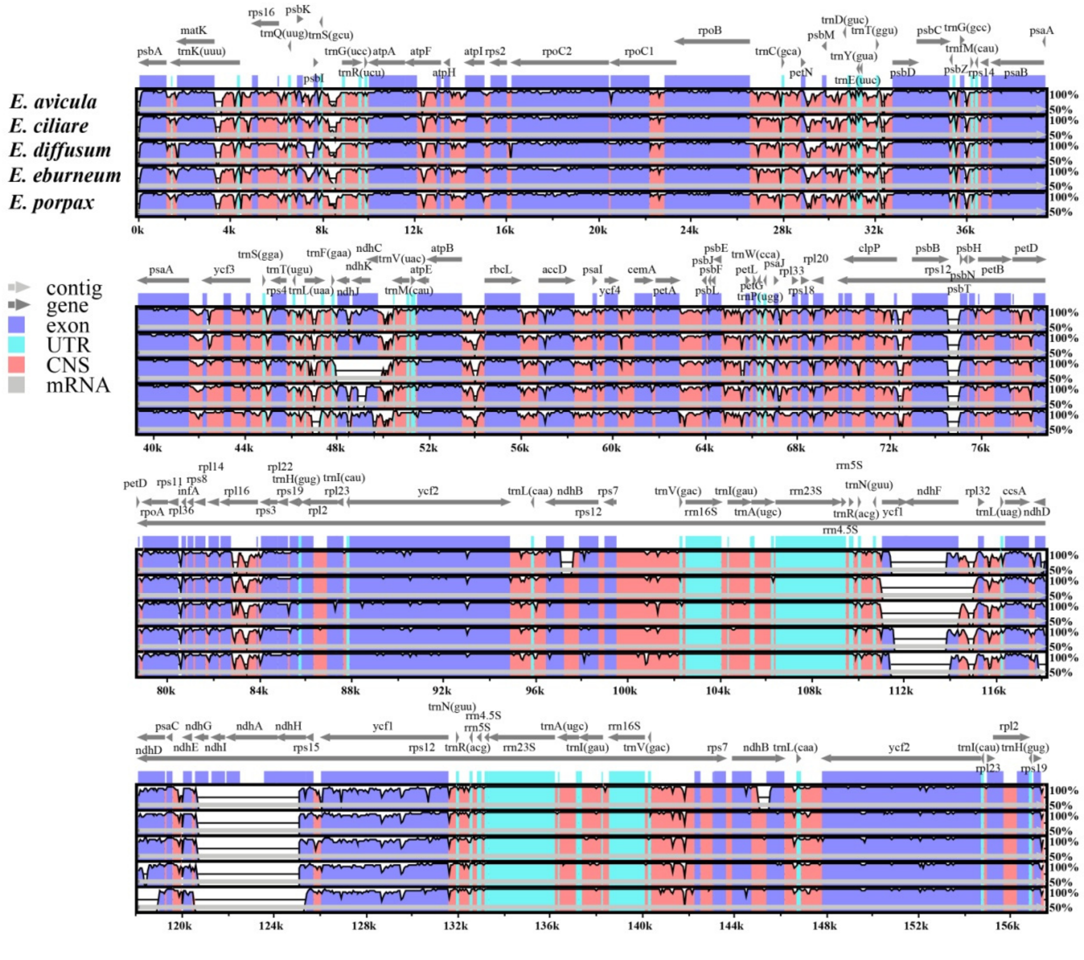

Supplementary Figure S2 Sequence alignment of complete plastomes of the five *Epidendrum*

species with *Stelis montserratii* as a reference. Thick, gray arrows above the alignment indicate the orientation and position of each gene. A cut-off of 70% identity was chosen for the plots. The Y-axis represents the identity percentage, ranging from 50 to 100%.

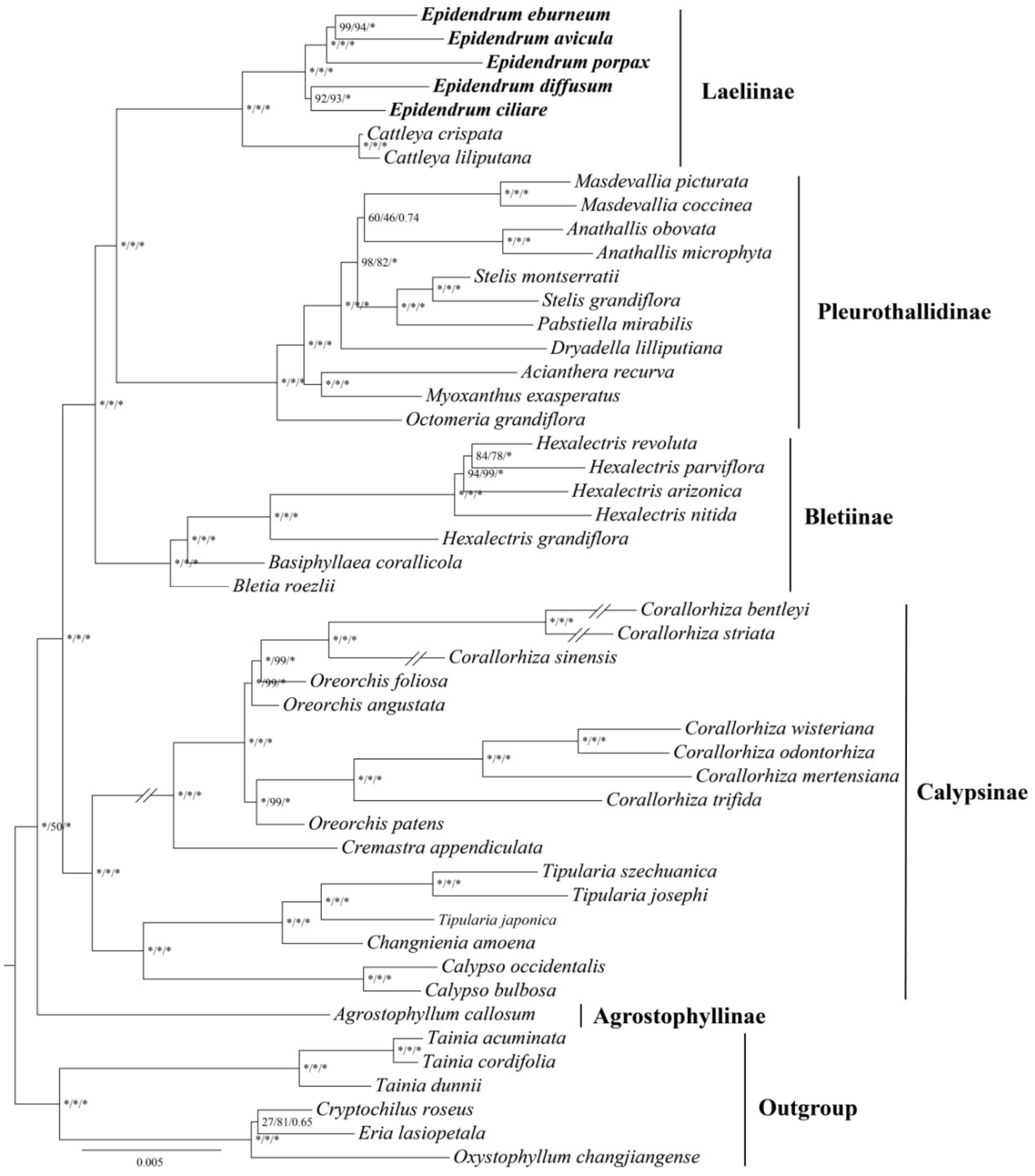

Supplementary Figure S3 The phylogenetic tree of 43 Epidendreae species obtained by maximum-likelihood analysis based on concatenated 68 protein-coding genes. The numbers near the nodes are bootstrap percentages and Bayesian posterior probabilities (BP_{ML}, BP_{MP}, PP), *node is 100 bootstrap percentage or 1.00 posterior probability.
